# Digital technology and human resource practices: A systematic literature review

**DOI:** 10.1016/j.heliyon.2025.e41946

**Published:** 2025-01-17

**Authors:** Robson Mekonnin Shiferaw, Zerihun Ayenew Birbirsa

**Affiliations:** a Haramaya University, College of Business and Economics, Department of Management, Ethiopia; b Jimma University, College of Business and Economics, Department of Management, Ethiopia

**Keywords:** Digital technology, Human resource, Practices

## Abstract

This research explores the relationship between digital technology and human resource practices, analyzing contemporary research conducted between 2016 and December 31, 2023. The systematic literature review adheres to fundamental review principles and identifies various studies conducted across various nations on the conceptual application of digital technology in human resource practices. This paper emphasizes the importance of understanding digital technology and human resource practices for all organizational levels, aiming to help professionals build and implement these practices for stronger competitive advantages in the digital technological era. Many methodological (exclusive search) constraints exist in this review, which may limit how well it can be used with additional case studies. Digital technology and human resource practices are essential parts of developing digital and advanced technologies relevant to human resource practices in many aspects. This systematic literature review analysed the relationship between digital technology and human resource practices, demonstrating their interconnectedness through a comprehensive review of research in this field. Both concepts of digital technology and human resource practices linkage was not more focused before. A full understanding of the current state of digital technology and research conceptualization for human resource practices on a single platform through developing a thorough technological concept in human resource practices.

## 1 Introduction

The use of digital technology in various business applications has altered how we work and how organisations manage their personnel [31]. Human resource practices specialists and academics are currently paying attention to contemporary digital technology such as cloud IT systems, artificial intelligence (AI), web platforms, and search engines because they are numerous, complex, impermanent, and highly interdependent on one another [40]. An increase in the use of automated, algorithmic workforce management approaches raises questions about their potential to reduce human bias and provide people who are engaged in algorithmic decision-making with a welcome and sustainable work environment [16].

Tracking technologies in human resource practices is increasing at a fast pace, and their duty at the workplace will become a standard soon. Many employees are currently working with technologies that allow for tracing employees' agility arrangements, behaviour, or even health conditions [1]. Technologies of human resource practices, such as electronic human resource practices, are assumed to strengthen the position of human resource practices as a business partner by promising strategic advantage [7]. Technological advancements in human resource practices permit the automation of every part of the job recruitment process, resulting in a highly automated employee interview system [35]. Development in data analytic technologies in human resource practices is claimed to support such developments in human resource technologies [39].

As discussed in the literature, digital technology-related aspects of human resource practices include digital technology-centred job autonomy, digital technology-centred job overload, and digital technology-centred job monitoring [37]. For institutions, electronic human resource practices offer cost advantages, capabilities, comparisons, and assurance. On the other hand, it is generally believed that using electronic human resource practices will result in higher-quality customer service [9].

The Internet of Things (IoT) has recently had a significant impact on human resources. 10.13039/100031019Digital technology provides a variety of facilities and support for human resource functionality like security, standards, privacy, and laws [41]. As an alternative to antiquated forecasting and succession planning approaches, a modern electronic human resource practices system allows matching with employees and appropriate job and career development [[49], [50]]. Digital technology-enabled electronic human resource practices capabilities and high-performance job system capabilities enhance organisations' human resource activities to the extent that these competencies create strategically coherent configurations [34].

As information Digital technology and best practices in human resource practices are highly prized resources, it seems sensible that combining these two areas of expertise would result in a stronger competitive edge [57]. More structured and effective innovation and technical improvement strategies taken into consideration and put into practice for businesses to maintain their operations and advance their technological development [17]. Only a few studies have specifically examined the relationship between technological innovation and human resources, even though technological innovation in human resource practices is increasingly becoming a highly demanding and vital driving force of institutional competitiveness [45]. The modern inclination towards the impact of technological improvements on both the assessment of engagement and the use of human resource practices and strategies to increase employee improvement [13].

Although artificial intelligence touted as a tool that will influence human resource practices, there is little academic study on how artificial intelligence can benefit these activities [43]. For cost and efficiency reasons in human resource practices, businesses are using artificial intelligence and algorithmic decision-making for their recruitment and selection processes. Nonetheless, there is a focus on the emotive response of job seekers to systems in the hiring process, and academics of human resource practices still need to comprehend the affective feedback to the selection process [31].

The increasing use of the internet and its applications, as well as the advent of digital platforms with human resource practices capabilities, have had a significant impact on the essential research topic of jobs and employment, which is undergoing significant changes [24]. Artificial intelligence in human resource practices has made it possible for employee recruiters to do their work daily and improved recruiting effectiveness at a fair price. Each stage of the hiring process, including hiring, promotions, job searches, applications, screening, assessments, and coordinating activities, benefits from the use of artificial intelligence [15].

This study analysed the boundaries of previously explored research relating to digital technology and human resource practices by critically assessing the chosen publications that were pertinent to the topic. Furthermore, the study tried to identify the link between digital technology and human resource, types of research design used and required for future scholars, differentiating commonly used research databases for future researchers, describing types of technologies applicable in human resource practices and where those technologies applied, and year of publication to check the sustainability of this concept.

Hence, we found that previously, scholars research studies had critical limitations pertinent to research methodology, geographical gap (i.e., more conducted in the western economy), and more of those selected research studies deployed mono method (quantitative, qualitative, mixed method separately in most of those articles selected for this review). As a result, we emphasised the limitations of earlier scholars studies related to this topic to pinpoint very essential shortcomings for future scholars to further consider and advance the link between digital technology and human resource practices in the future.

## 2 Conceptualization of digital technology and human resource practices

The relationship between digital technology and human resource practices. The impact of digital technology systems on company performance demonstrated by adaptive structuration theory and embeddedness theory [60]. In the context of the private banking component, organisational trust and technological adaptation for human resource practices are methods based on social exchange theory [51]. Organisations become knowledge organisations that can fulfil the demands of tailored training and enhance the quality of learning in human resource practices thanks to emerging concepts of artificial intelligence-based training [14]. Questions about the fairness of decisions made by artificial intelligence in human resource practices whether or not employees receive respectful treatment, such as interactional justice, may arise [6].

By utilising telecommuting as a cutting-edge digital technology in contemporary human resource practices, telework-oriented human resource practices primarily affect teleworkers' happiness and well-being via stress [22]. Workplace circumstances in businesses have changed because of the increased use of digital technology in recent years. The retail industry, for instance, has benefited from the development and widespread adoption of electronic commerce technologies [2]. Workplace improvement mostly driven by a few key reasons, including the use of human resource practices, digital technology, artificial intelligence in human resources, automation of human resources, changes to workplace policies that affect human resources, and the expanding labour market [3].

Rhetoric predicting that technical advancements like the use of electronic human resource practices and digital technology will make human resource practices more strategically focused is accompanying the development of internet-based human resource practices [38]. It is easier to understand the benefits and drawbacks of many stakeholders relevant to the junction of human resource practices and digital technology when human resource practice principles applied to information, digital technology, and human resource practices [10]. Developing and using electronic human resource practices systems that could assist businesses' workflow processes and allow employees to do a variety of human resources and other human resource functions more successfully [26]. There is currently a rise in the use of artificial intelligence tools and digital analytics to create and retain competitive advantage through strategic and dynamic capabilities [42].

Exploring how digital technology is used in human resource practices, in particular, how electronic human resource practices are used and how it affects employee productivity, is made easier with the help of social exchange theory [25]. In contrast, this analysis examines the viewpoint and expectations of human resource practices experts and operational managers concerning new roles they can play in a scenario where a set of digital technologies applied to the discipline [27]. Conversely, attempts made to conceptualise digital technology in human resources as a nexus of practices and their factual structure, but with little theoretical development in electronic human resource practices in academic research [21].

Based on the fundamental flaws of past reviews and research articles written by academics with various conceptual levels, this study intended to address the following critical review problems.

### 2.1 Review questions

Q1 How the relationship between digital technology and human resource practices addressed?
Q2 What types of research approaches used in those articles to collect research data?
Q3 In which databases journal articles of scholars published?
Q4 What are the types of technologies researchers used in their research study regarding human resource practices?
Q5 Where are digital technology and human resource practices in countries conducted?
Q6 In which year did scholars investigate mostly digital technology and human resource practices?

## 3 Research method

This study used a methodical review of the literature to find papers that define or conceptualise the idea of digital technology and practices for human resource practices. We chose the systematic review of the literature over narrative and meta-analysis because of their advantages. The social sciences have adapted some of this approach's characteristics. To assess whether the method is appropriate for the discipline of human resource practices, it has recently undergone extensive scrutiny. By compiling information from several studies, a systematic review aids in the creation of a trustworthy knowledge base. This study looks at how these ideas differ as well as what they have in common, which is included in this review. Systematic literature reviews and non-systematic literature reviews (non-SLRs) are the two categories of literature reviews that can be considered autonomous studies. It is crucial to understand that SLRs and non-SLRs are review types rather than review nomenclatures, names, or naming [32]. On the other hand, there are various types of systematic review papers: structured review that concentrates on commonly used theories, methods, and constructs [20].

### 3.1 Search strategy

It was feasible to establish transparent, well-documented research practices with criteria for article inclusion and exclusion thanks to the methodical identification of relevant research papers. The systematic review process involves several steps, including formulating research questions, deciding on inclusion and exclusion criteria, setting standards for gathering literature, developing a comprehensive search strategy, developing a codebook for categorising and describing literature, coding the literature, and synthesising the literature.

This review addresses the many ways that digital technology and human resource practices studied in researchers' studies by looking at the literature to see if the ideas of digital technology and human resource practices have a good relationship. The search approach largely used to locate research publications that integrate digital technology with best practices in human resource management. The review's inclusion and exclusion criteria developed in order to achieve this.

### 3.2 Sample selection process

Research publications found in the initial search, 456 of which were in English.

**Fig. 1:**
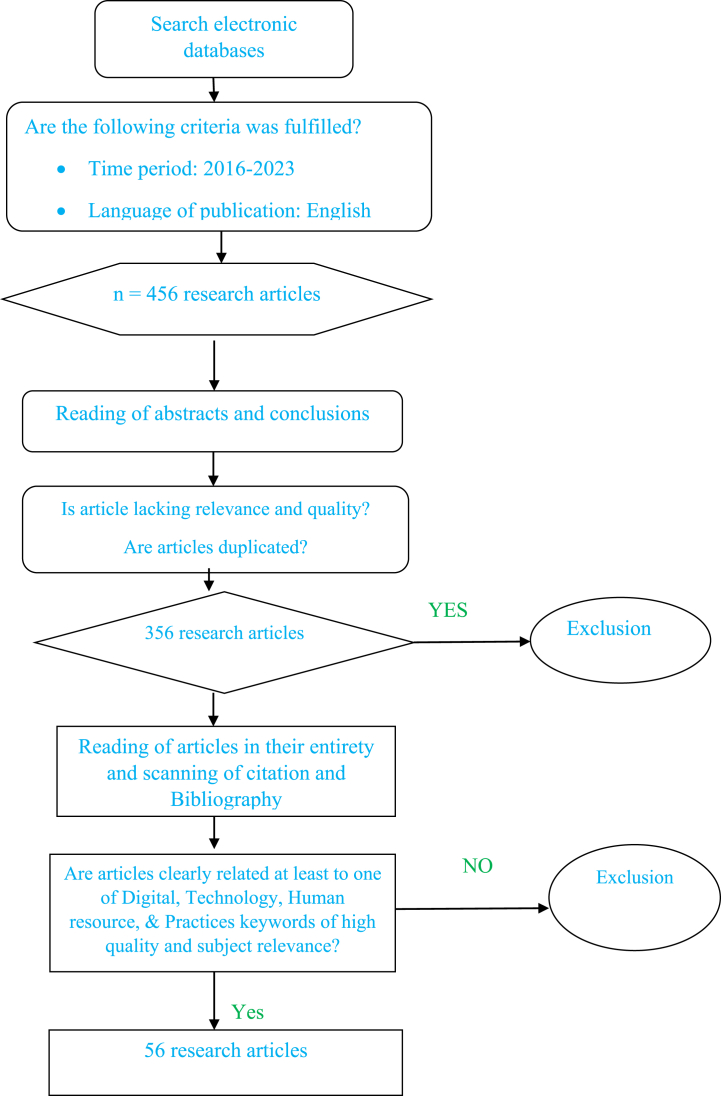
Article selection (process using PRISMA).

**Source**: Adapted from Ref. [36].

The selection process flow diagram shown in broad strokes in Fig. 1.

#### 3.2.1 Selection of databases

To find scholarly research publications, the study used a three-step process. First, we chose important databases for business, management, and social science. To accomplish the review's goal, articles about digital technology and best in human resource practices sought out using seven major academic databases that are Scopus-indexed, including Emerald Insight, Taylor & Francis, Springer, Sage, Wiley Online Library, and Science Direct. So, the databases were chosen primarily based on the topic's characteristics (digital technology and human resource practices) by assessing each article downloaded using a methodical literature review approach.

#### 3.2.2 Inclusion and exclusion criteria

Inclusion and exclusion criteria used to conduct the review. The search variable set, publication year, language, and search boundary make up the inclusion criteria. The social sciences, management, human resource practices, and management journals undergone peer review and are widely respected were the search boundaries. The search practices concentrated on academic articles published between 2016 and December 31, 2023.

To publish and further alter the paradigm in terms of digital technology and human resource management methods, the year 2016 chosen as the baseline for the earliest date of interest. English-language research publications were the only ones that were included in the study search. The study used search engines utilising the Boolean approach and employing search terms on "digital technology," human resource practices, "electronic human resource practices," and "innovation.”

Relevance, excellence, and duplication are the exclusion criteria. This achieved by carefully analyzing the abstracts and conclusions of the downloaded article databases used in this investigation. To improve the findings of this review, the study excluded unpublished articles, article contents, book reviews, miscellaneous books, book chapters, working paper series, and conference papers. The relevance of the articles was determined by determining whether they fit a keyword used as a search string. By assigning codes to each article and using manual detection, duplicate articles eliminated.

Following a thorough screening process by the researchers, we established a publication pool of 456 articles after identifying and eliminating duplicates. Using several inclusion and exclusion criteria, we screened these papers. Selected articles in total 56 met the inclusion and exclusion criteria. A systematic, reliable, replicable, and transparent approach to data gathering and analysis of the paper used during article selection for a systematic review created by the review practices.

### 3.3 Data analysis

Concerning review questions that were predetermined at the first stage of the review steps, we applied a descriptive and content analysis. Using the data mining form's categories, a descriptive analysis carried out. To do this, the review matrix was tabulated, especially for the discussion of research feature findings utilising points such as databases, kind (conceptual, empirical, or review), and level of analysis. The descriptive analysis aids the research characteristics subsection and gives the reader a quick overview of the reviewed publications in this study. In addition, content analysis employed as a data analysis practice. The publications addressed encoding-related topics, and a manual interpretative approach employed to examine the study's outcomes. The first paragraph of this section provides an overview of the 56 identified journal articles' case topics, contributions, constraints, research methodologies, and distribution by years and journals. The next section goes into detail on how digital technology and human resource practices have been conceptualised by past researchers.

## 4 Results and discussions

This section begins by outlining the conceptual applications of the 56 chosen journal research articles in terms of digital technology, human resource practices, journal based distribution, year based distribution, country based distribution, database based distribution, and research approach based distribution. The study used for interpretation and discussion purpose sense making process that is, scanning, sensing, and substantiating each crucial to deriving meaningful interpretations for this study as stated in the work of [61].

The next section highlights the digital technology and human resource practices concepts put forth by earlier research investigations.

### 4.1 Results

#### 4.1.1 Country based distribution of articles

Among the 56 research articles chosen, the distribution of publications by nation shown (See Appendix A). Eight out of the 56 articles chosen, or the majority of digital technology and human resource practices undertaken in the United States. Comparatively speaking, China and Germany each account for six research articles on the given issue. The United Kingdom and the Netherlands rank five and four, respectively, on digital technology human resource practices. Moreover, Australia, Bangladesh, and Canada each provide three articles, respectively. In addition, two papers on digital technology and human resource practices undertaken in each of the following nations: Sweden, Spain, Pakistan, India, and Pakistan. Lastly, Belgium, Finland, Greece, Italy, Nigeria, the Philippines, South Africa, Scandinavia, and South Korea published one article in each country, respectively. As a result, we conclude that research on digital technology and human resource practices within the specified duration is less prevalent in Africa, the Middle East, and Eastern Europe. This could suggest a regional need for future researchers to consider when providing information on digital technology and human resource practices.

#### 4.1.2 Types of research approaches

The 56 research articles about digital technology and human resource procedures selected for a systematic review and published in different journals are depicted (See Appendix B). The analysis indicated the proportion of the 56 research publications that employed the different research types or methods (as previously described) to advance the interests of practitioners and the scientific community. A quantitative research strategy accounts for 48 % of the studies of prior researchers' research approaches on digital technology and human resource practices. In addition, the second study methodology used by academics, accounting for 38 %, was qualitative. While a few of them (18 %) used a systematic review, 2 % used a mixed method, and 2 % used bibliometric analysis. We conclude that researchers who focused on digital technology and human resource practices less triangulated in their research approach.

#### 4.1.3 Year based distribution of articles

To discover trends in digital technology and human resource practices research, academic research articles grouped according to the years they published, from 2016 to 2023. The number of publications on digital technology and human resource practices started to rise in 2016 and 2017, fell by half in 2018, and then started to rise again in 2019 (See Appendix C) illustrates. Nonetheless, the picture shows that from 2020 onwards, there is an exponential increase as well as a tendency for falling growth in 2023. This suggests that, in various corporate circumstances, there is a need to increase intent and interest related to digital technology and human resource management practices.

#### 4.1.4 Research databases

This study determined the most popular research databases that academics favoured between 2016 and 2023 (See Appendix D). The bulk of academic researchers' research papers published using Emerald Insights, which accounts for 12 out of 56 research papers chosen among the research databases. The Wiley Online Library and Taylor and Francis databases, which each account for 10 research papers during a specific period relevant to digital technology and human resource practices, are the second and third best research databases, respectively. Yet, within the periods outlined in this review, scholars accessed the same number of Springer and Sage databases. In addition, the scholars on the subject who were identified for their.

#### 4.1.5 Types of technologies in human resource practices

The distribution of 56 selected research articles by categories of digital technology applications in human resource practices shown (See Appendix E). Electronic human resource practice technologies used in the majority of digital technology and human resource practices, or 14 out of the 56 publications chosen. In contrast, artificial intelligence in human resources and fuzzy TISM digitization were the focus of 13 and 12 research articles on the topic, respectively. Information technology and human resource digitalization make up 5 and 4 of those challenges, respectively. Two articles, out of 56 total, written about the use of performance management digital technology in human resource practices.

Finally, one article on each of the following: digital technology at work, smart technologies, robotics at work, real-time monitoring, digital tools, and automated employee interviews with digital technology was released. As a result, we conclude that the majority of research articles on the use of digital technology in human resource practices are relevant to concerns involving employees. Future researchers urged to see the limitations of the work of earlier academics because of technological advancements like robotics, automated interviewing, and real-time monitoring.

#### 4.1.6 Journal based distribution of articles

This description made to track the publication locations of digital technology and human management practices research articles in journals. From 2016 to 2023, 56 highly reputable peer-reviewed, Scopus-indexed publications published research articles on digital technology and human resource practices under various headings (See Appendix F). Academicians who are concerned about finding and deciding on a publication for articles on digital technology and human resource practice strategies in the future may find this statistic encouraging. Out of these reputable publications, the International Journal of Human Resource Practices research was the one that predominated in accepting and publishing articles on digital technology and human resource practices.

### 4.2 Discussions

The trend towards utilising digital technology in human resource practices brought about, for example, by the fact that robots, as opposed to human labour, do not contract the coronavirus [11]. In addition, by providing technological tools, it is possible to increase employee happiness and maybe build a solid employer brand and reputation [[4], [55]]. An employee may receive favourable feedback regarding the choice of valence than unfavourable input, which often results in a greater perception of polite behavior. They preferred good artificial intelligence outcomes to negative human feedback on various indications, for example, where these circumstances cause possible conflict [6]. Currently, it believed that artificial intelligence is necessary for carrying out operational tasks, notably in the field of human resource practices. Yet in reality, the rate of adoption of these modern algorithms by businesses is still in its infancy [28]. Developing artificial intelligence practices that can affect the majority of human resource practices, as well as their potential to increase demand for contemporary technologies [29].

Therefore, it is difficult to make long-term, accurate predictions regarding how advanced digital technology will affect corporate sectors, communities, and labour affairs. Research has always focused on examining new technologies, labour, and working settings [[8], [12]].

Digital technology and digitalization have had an impact on several service sectors. Even knowledge-intensive business-to-business services are in high demand because of the combination of digital technology and human resource practices [[46], [59]].

The ability of employees to embrace newly introduced digital technology for human resource practices in business enterprises influenced by ways to participate in decision-making [51]. Electronic human resource practices have become an important academic and administrative concern because of technological advancements [44]. A paradigm shift in how we can work brought about by modern firms' tremendous improvements in the usage and accessibility of digital technology in human resource practices [23]. Worker innovation within an organisation's human resource practices can increase labour productivity [54].

There has been much debate over how to construct concepts and research streams regarding to proper conceptualise the content of human resource information systems, virtual human resources, electronic human resource practices, and business-to-employee systems separately and comprehensively over many years of academic and human resource experts' discourse on human resource technologies [[18], [30]]. For instance, the top management's support for digital technology-based human resource practices and the atmosphere for human resource practice innovation better optimise portfolios to maximum satisfaction [19].

To monitor interest-based job distribution, activity allocation, and access to equal opportunities for all, detailed reporting of a worker's current and prospective job assignments uses digital technology development [[33], [52]]. Multinational companies quickly responded to these circumstances by creating digital solutions for all business operations. The technological revolution of human resource practices thought to have prompted institutional ambidexterity [[47], [53]]. The extent to which artificial intelligence used in human resource practices, as well as the conditions that already exist and its potential future effects on human resource practices [48]. Digital technology-based human resource practices have advanced significantly, as digitalization disrupts repetitive practices [58].

Uncertainty exists on how to combine stakeholder acceptability of artificial intelligence with enterprises' and key beliefs about the processes and results of digital creativity [56]. Modern work has undergone a digital transition because of the technological advancements in human resource practices, which have sparked a new industrial revolution. The worldwide COVID-19 epidemic has accelerated digital technology in human resource practices of their business operations [5]. Social exchange disrupted by artificial intelligence and the research on decision-making and choice shows that utilising artificial intelligence, and human resource practices in decision-making produced superior results in terms of understanding and accountability [49].

## 5 Practical implications

This study has significant conceptual contributions to the scientific community and practitioners. The study offers a comprehensive, systematic review of 56 identified research articles in management and human resource practices to reveal how scholars have explored this concept so far and present a route for future research agendas. Besides, this study contributes to the basic knowledge of digital technology and human resource practices after 2016. Mainly, this study contributes by identifying the relationship between digital technology and human resource practices as well as the underlying views about the application of digital technology pertinent to human resource practices in different contexts. Reviewing key insights from existing scholars' conceptualizations of the paradigm and supporting that the conceptual foundations have derived brief definitions, broad in scope and views, derived to advance empirical research on digital technology and human resource practices. This research thought valuable source for researchers in different countries to understand the present circumstances pertinent to the application of digital technology and human resource practices. On the other hand, in the field of human resource practices and management for future stakeholder engagements, from the traditional manners to digital ways of performing human resource practices functions such as electronic human resource practices and artificial intelligence to alleviate partiality during the recruitment and selection process.

For professionals, this research demonstrates the favourable outcomes organisations can derive by having a proper understanding of digital technology and human resource practices' conceptual application. Implementing digital technology applications in human resource practice in a firm's strategic plan plays a vital role in gaining a competitive advantage because of the digital transformation of the organization. Thus, digital technology application in human resource practices plays an imperative role in that concern; this review will help firms comprehend that.

## 6 Conclusion

The objective of this systematic review was to review contemporary literature on digital technology and human resource practices' conceptual applications from different scholars' perspectives from 2016 to 2023. Digital technology and human resource practices conceptual applications investigated within a given period specified in most developing and less developed countries. Most of the studies focused on the digital technology side rather than the human resource practices. This type of disintegration can be misleading, and a systematic review provides a useful analysis to highlight the disintegration and propose boundaries to investigate digital technology and human resource practices' in their business firms.

## 7 Limitations of this review

However, a systematic review also has methodological limitations, including the level of accuracy and preferences not being the same with different scholars. To overcome this type of limitation, we focused on top-known research databases trusted broadly by scholars pertinent to the discipline and the topic of the issue under review. Even if some scopes might missed, we trust that our conclusion found a reasonable acceptance of redundancy in the databases that we used for this study due to our focus only on eight years. We recommend that future researchers investigate by relaxing the time and considering the geographical gap identified in this study. Specifically, a future researcher may contribute to the literature older than this study and clarify our findings. Importantly, the findings of this review confirm that, despite the relatively established body of literature, there is a lack of direct application between digital technology and human resource practices in most contexts. There is no mutual agreement among human resource practices scholars and practitioners about the exact relationships between digital technology and human resource practices concepts, but rather the associates with different business orientations.

## 8 Future researchers’ agenda

A future researcher, in particular, may contribute to the literature older than this study and clarify our findings. Importantly, the review's findings reveal that, despite a somewhat established body of literature, there is a paucity of direct application of digital technology and human resource practices in most scenarios. Human resource scholars and practitioners do not agree on the precise linkages between digital technology and human resource practices principles but rather associate with distinct business views.

## CRediT authorship contribution statement

**Robson Mekonnin Shiferaw:** Writing – review & editing, Writing – original draft, Resources, Methodology, Investigation, Formal analysis, Data curation, Conceptualization. **Zerihun Ayenew Birbirsa:** Visualization, Validation, Supervision, Resources.

## Ethics approval

This study systematic literature review using PRISMA checklist in accordance with the study scope. The checklist was attached with this article manuscript.

## Abbreviations

Not applicable in this document.

## Data availability statement

Data will be available.

## Additional information

Additional information is available.

## Funding

We declare that there is no fund received in publishing this paper.

## Declaration of competing interest

The authors declare the following financial interests/personal relationships which may be considered as potential competing interests: Robson Mekonnin Shiferaw reports administrative support was provided by 10.13039/501100005068Jimma University College of Business and Economics. The authors declare their is no conflict of interest in financial or professional issues If there are other authors, they declare that they have no known competing financial interests or personal relationships that could have appeared to influence the work reported in this paper.

## Figures and Tables

**Figure:**
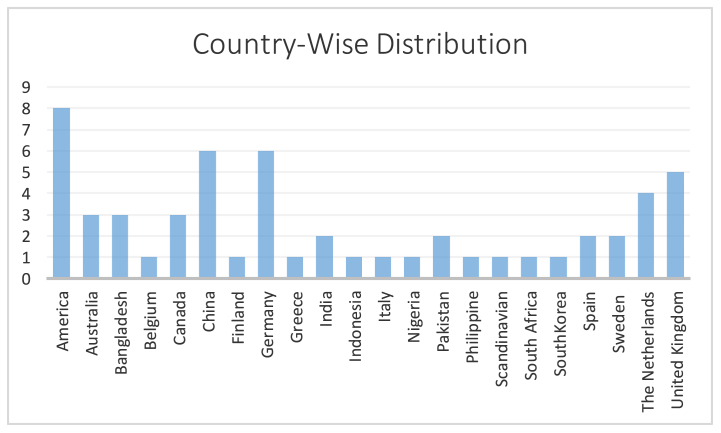
Appendix ADistribution of articles across countries

**Appendix B:**
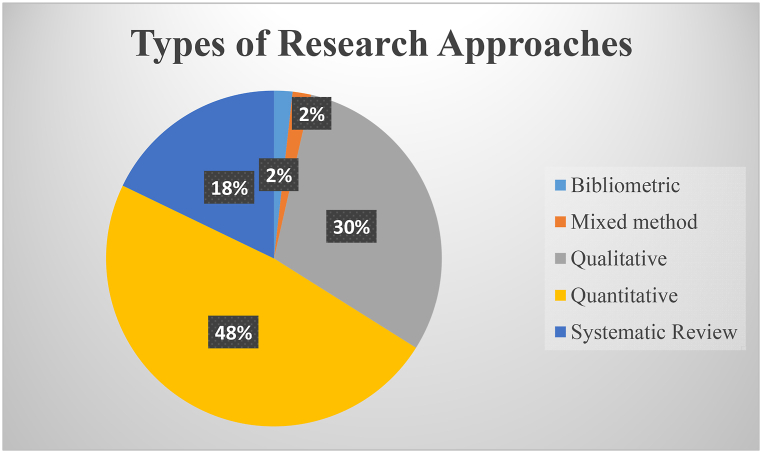
Types of research approaches

**Appendix C:**
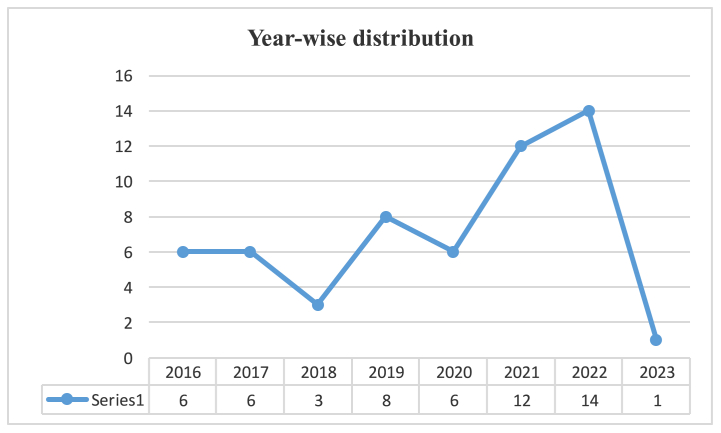
Year based distribution of articles

**Appendix D:**
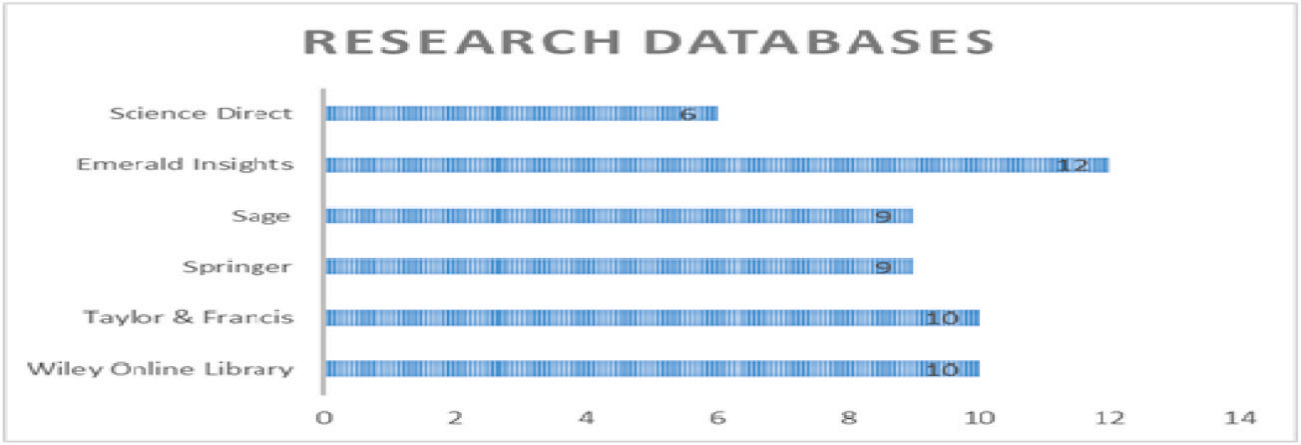
Research databases

**Appendix E:**
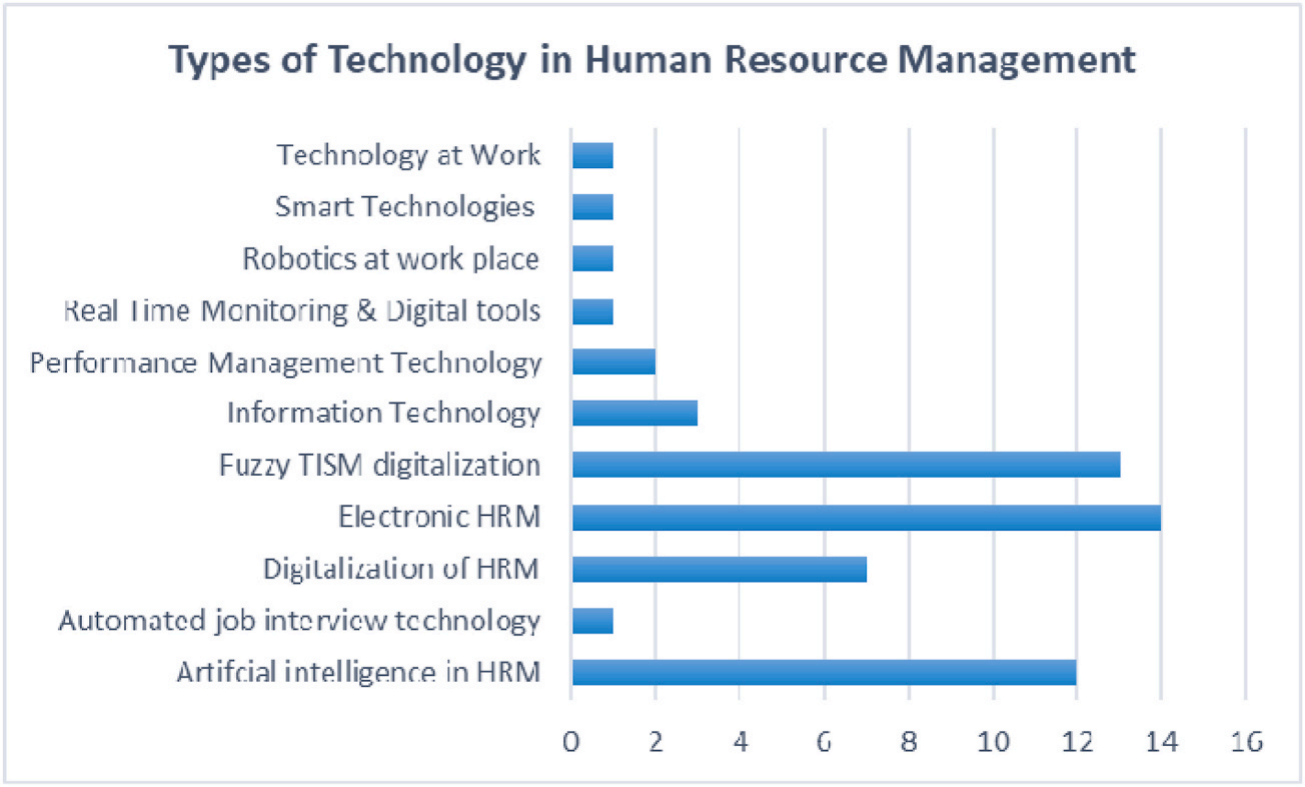
Types of technology in human resource management.

**Table:** 

S.No	List of Journals	Number
1	Advanced Series in Management	1
2	Asia Pacific Journal of Human Resources	1
3	Asian Journal Of Digital technology Innovation	1
4	Behaviour & Information Digital technology	1
5	Canadian Journal of Administrative Sciences	1
6	Cognition, Digital technology & Work	1
7	Electronic Journal of Information Systems in Developing Countries	1
8	Electronic Human Resource Management in the Smart Era	1
9	Employee Relations: The International Journal	2
10	Enterprise Information Systems	1
11	European Surgery	1
12	German Journal of Human resource practices	1
13	Human Resource Journal	1
14	Human resource practices Review	2
15	Human-Centred Organisations Advanced Series in Management	3
16	Information Systems Frontiers	2
17	International Journal Select Assess	1
18	Intelligent Systems in Accounting, Finance and Management	1
19	International Journal of Information Management Data Insights	1
20	International Journal of Productivity and Performance Management	1
21	International Studies of Management & Organization	1
22	Journal of Enterprise Information Management	1
23	Journal of Innovation & Knowledge	1
24	Journal of International Business Studies	1
25	Journal of Service Research	1
26	Journal of Small Business & Entrepreneurship	1
27	Journal of the Knowledge Economy	1
28	Management Decision	2
29	Management Research Review	1
30	New Digital technology, Work, and Employment	1
31	National Human Resource Development Network	1
32	Public Personnel Management	1
33	Research in Personnel and Human Resources Management	1
34	Review of Managerial Science	2
35	SAGE Open Journal	1
36	Software: Practice and Experience	1
37	South Asian Journal of Human Resources Management	2
38	The International Journal of Human Resource Management	1
39	Technological Forecasting & Social Change	1
40	The Economic and Labour Relations Review	1
41	The International Journal of Human resource practices	8
